# Endothelial SUR-8 Acts in an ERK-Independent Pathway During Atrioventricular Cushion Development

**DOI:** 10.1002/dvdy.22343

**Published:** 2010-06-14

**Authors:** Jing Yi, Muyun Chen, Xiaohui Wu, Xiao Yang, Tian Xu, Yuan Zhuang, Min Han, Rener Xu

**Affiliations:** 1 Institute of Developmental Biology & Molecular Medicine, School of Life Sciences, Fudan UniversityShanghai, PR China; 2 Genetic Laboratory of Development and Diseases, Institute of BiotechnologyBeijing, PR China; 3 Howard Hughes Medical Institute and Department of Genetics, Yale University School of MedicineNew Haven, Connecticut; 4 Department of Immunology, Duke University Medical CenterDurham, North Carolina; 5 Howard Hughes Medical Institute and Department of MCDB, University of ColoradoBoulder, Colorado

**Keywords:** SUR-8, atrioventricular cushion, MAPK/ERK, congenital heart disease, endothelial-mesenchymal transformation (EMT)

## Abstract

SUR-8, a conserved leucine-rich repeats protein, was first identified as a positive regulator of Ras pathway in *Caenorhabditis elegans*. Biochemical studies indicated that SUR-8 interacts with Ras and Raf, leading to the elevated ERK activity. However, the physiological role of SUR-8 during mammalian development remains unclear. Here we found that germline deletion of SUR-8 in mice resulted in early embryonic lethality. Inactivated SUR-8 specifically in mouse endothelial cells (ECs) revealed that SUR-8 is essential for embryonic heart development. SUR-8 deficiency in ECs resulted in late embryonic lethality, and the mutant mice displayed multiple cardiac defects. The reduced endothelial-mesenchymal transformation (EMT) and the reduced mesenchyme proliferation phase were observed in the atrioventricular canal (AVC) within the mutant hearts, leading to the formation of hypoplastic endocardial cushions. However, ERK activation did not appear to be affected in mutant ECs, suggesting that SUR-8 may act in an ERK-independent pathway to regulate AVC development. Developmental Dynamics 239:2005–2013, 2010 © 2010 Wiley-Liss, Inc.

## INTRODUCTION

Congenital cardiovascular malformation is one of the most common anomalies (Hoffman,^1995^; Hoffman and Kaplan,^2002^), and maldevelopment of septation and valvulogenesis accounts for about one quarter of all cardiovascular malformations (Loffredo,^2000^). Vertebrate valvulogenesis is a complex process, initiated by a subset of endothelial cells specified to undergo a critical endothelial-mesenchymal transformation (EMT) and invade into the extracellular matrix to form endocardial cushions. The cushion mesenchymal cells then undergo a proliferation phase and the final remodeling process to form mature thinly tapered valves (Armstrong and Bischoff,^2004^; Person et al.,^2005^).

The EMT process is commonly measured in vitro by a three-dimensional collagen gel assay (Runyan and Markwald,^1983^; Camenisch et al.,^2002^), and multiple signaling pathways have been documented to be involved in EMT. For example, Notch signaling is required for the endothelial cell capacity for mesenchymal cell fate (Timmerman et al.,^2004^). TGF-β signaling synergizes with BMP signaling to facilitate EMT (Nakajima et al.,^2000^; Delot,^2003^), whereas either more or less VEGF doses cause EMT defects (Miquerol et al.,^2000^; Dor et al.,^2001^). In addition, Ras signaling has been shown to regulate EMT in both human congenital valve malformations and Ras pathway disruption in animal models (Yutzey et al.,^2005^). However, the regulatory networks underlying cardiac valve development, particularly EMT, are still not well understood, and novel factors important for valve development remain to be identified (Combs and Yutzey,^2009^).

SUR-8 was first identified as a positive regulator of the EGFR/Ras pathway in *C. elegans* in a genetic screening for the suppressor of activated Ras during vulval induction (Sieburth et al.,^1998^). SUR-8 was also independently identified to act downstream of FGFR pathway in *C. elegans* (Selfors et al.,^1998^). SUR-8, in both *C. elegans* and mammals, was shown to directly interact with the Ras effector domain (Sieburth et al.,^1998^). The mammalian SUR-8 was also found to be able to form a ternary complex with Ras and Raf, specifically enhancing the Ras-induced ERK activation without any effect on AKT or JNK activity (Li et al.,^2000^). Further evidence showed that SUR-8 acted as a specific M-Ras effector by recruiting the catalytic subunit of protein phosphatase 1 (PP1c) to modulate Raf activity (Rodriguez-Viciana et al.,^2006^). Erbin, a negative regulator of Ras pathway, was shown to regulate the Raf activity by attenuating the interactions of SUR-8 with Ras and Raf (Huang et al.,^2003^; Dai et al.,^2006^). In contrast, SUR-8 sh-RNA knockdown analysis in *Drosophila* S2 cell line suggested that SUR-8 did not contribute to Raf activation in response to stimuli (Anselmo et al.,^2002^). Moreover, SUR-8 deficiency alone in *C. elegans* did not cause any obvious defect, indicating that SUR-8 is not essential for the normal development in the worm. However, the physiological significance of mammalian SUR-8, which is expressed in multiple tissues in both human and mice (Selfors et al.,^1998^; Su et al.,^2004^), has yet to be explored.

To investigate the physiological function of SUR-8 in the mammalian cardiovascular system, we generated mice with SUR-8 deletion specifically in endothelial cells by using Cre-LoxP system. We found that SUR-8 inactivation in the cardiovascular system led to late embryonic lethality in mice with multiple cardiac defects, indicating that SUR-8 plays an essential role in mouse embryonic heart development.

## RESULTS

### Inactivation of SUR-8 in the Endothelial Cells Leads to Embryonic Lethality

**Fig. 1:**
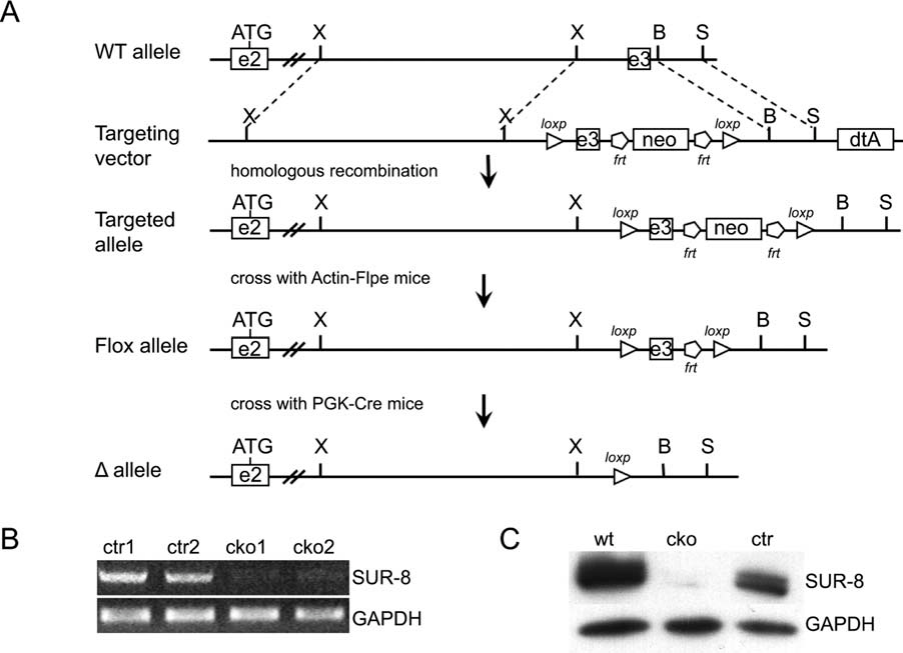
Conditional inactivation of SUR-8 in endothelial cells. **A:** Schematic illustration of mouse *Sur-8* wild-type allele, targeting vector and the *loxP* modified *Sur-8* loci. Breeding the mice containing the targeted allele with Actin-Flpe transgenic mice results in pups with the flox allele. The disrupted (Δ) allele was then obtained by crossing the flox mice with PGK-Cre mice. Restriction enzyme sites: X, XbaI; B, BglII; and S, SpeI. **B:** RT-PCR analysis of isolated endothelial cells using primers specific to *Sur-8* gene. GAPDH was used as an internal control. **C:** Western blotting analysis with an anti-SUR-8 antibody on isolated endothelial cells derived from different genotypes. GAPDH was used as an internal control. wt, *Sur-8*^+/flox^; cko, *Sur-8*^Δ/flox^;Tie2-Cre; ctr, *Sur-8*^Δ/flox^.

We initially made conventional knockout mice for the *Sur-8* gene, resulting in embryonic lethality at an early stage, and partial absorption of mutant embryos at E8.5 (data not shown). To examine SUR-8 function in specific tissues and at later stages of mouse development, Cre-loxP system was used for conditional disruption of the *Sur-8* gene. Briefly, our vector contained two *loxP* sites inserted to flank the *Sur-8* exon 3, followed by a neomycin resistance cassette flanked by two *frt* sites (Fig. 1A). Heterozygous mice with the targeted allele (*Sur-8*^flox-neo/+^) were crossed with Actin-FLPe transgenic mice (Jackson Laboratory, Bar Harbor, ME) to remove the neomycin resistance cassette, which may affect the expression of the targeted gene (Chen et al.,^1993^). The offspring *Sur-8*^flox/+^ were then crossed with PGK-Cre mice to obtain mice with one of the *Sur-8* alleles disrupted (*Sur-8*^Δ/+^). *Sur-8*^Δ/Δ^ embryos from *Sur-8*^Δ/+^ intercrossing also displayed the early-stage lethality, similar to the homozygous embryos derived from the *Sur-8* conventional knockout mice (data not show). Meanwhile, mating between heterozygous *Sur-8*^flox/+^ mice yielded homozygous floxed *Sur-8* (*Sur-8*^flox/flox^) mice at the expected Mendelian ratio. These floxed mice were viable, fertile, and did not show any gross abnormalities, and thus are suitable for examining the effect of the conditional inactivation of SUR-8.

To specifically disrupt the *Sur-8* gene in endothelial cells, the floxed *Sur-8* mice were bred with Tie2-Cre transgenic mice, in which Cre recombinase expression was driven by the Tie2 endothelial-specific promoter (Lan et al.,^2007^). Reverse transcription (RT)-PCR and Western blot analysis confirmed the efficient inactivation of SUR-8 in the endothelial cells derived from *SUR-8*^Δ/flox^; Tie2Cre embryos (Fig. 1B and C). Of 87 viable offspring analyzed from the crosses between *Sur-8*^Δ/+^;Tie2-Cre males and *Sur-8*^flox/flox^ females, we obtained no *Sur-8*^Δ/flox^;Tie2-Cre (designated as conditional *Sur-8* knockout [CKO]) progeny, and the offspring numbers with the other three genotypes were very close to the expected Mendelian ratio (Table 1), indicating that EC-specific deletion of SUR-8 led to embryonic lethality.

**TABLE 1:** Viability of Progeny from Crosses between *Sur-8*^*flox/flox*^ and *Sur-8*^Δ/+^; *Tie2-Cre* Mice

Genotype	E12.5	E13.5	E14.5	E15.5	Birth
*Sur-8*^+/*flox*^	13	28	15	11	31
*Sur-8*^Δ/*flox*^	17	32	24	10	27
*Sur-8^+/flox^; Tie2-Cre*	10	27	25	7	29
*Sur-8^Δ/flox^; Tie2-Cre*	12 (0 dead)	28 (2 dead)	19 (5 dead)	8 (8 dead)	0
#Litter	6	12	9	4	11

Viability was determined by the presence of visible heartbeats.

Embryos at different developmental stages were examined to determine the stage when the *Sur-8* CKO embryos died (Table 1). By E11.5, the *Sur-8* CKO embryos were viable, determined by the presence of visible heartbeats, and morphologically indistinguishable from their wild-type littermates (data not shown). However, at E12.5, the *Sur-8* CKO embryos began to exhibit some sporadic hemorrhaging in the surface (data not shown). The *Sur-8* CKO embryos displayed smaller body size at E13.5, and half of them showed subcutaneous edema in their dorsal body and fetal lung congestion at E14.5 (see Supp. Fig. S1A and B, which is available online). No viable *Sur-8* CKO embryos could be obtained at E15.5.

### *Sur-8* CKO Embryos Exhibit Multiple Cardiac Defects

To examine the possible cause of embryonic lethality, we performed the histological analysis on the E13.5 *Sur-8* CKO placenta, and comparable placental size and structure were observed between the mutants and controls (data not shown), excluding the possibility that embryonic demise was caused by extraembryonic vascular defect. We also examined the peripheral vessel network and embryonic vessel maturation. The whole-mount antibody staining of PECAM-1, a receptor highly expressed on endothelial cells, showed the similar vascular patterns of the *Sur-8* CKO embryos as that of the controls (Supp. Fig. S1C). H&E staining of transverse sections of dorsal aorta from the *Sur-8* CKO embryos showed that endothelial cells are normally surrounded by vascular smooth muscle cells (data not shown), suggesting that SUR-8 deficiency may not affect blood vessel maturation and integrity during mouse embryonic development.

**Fig. 2:**
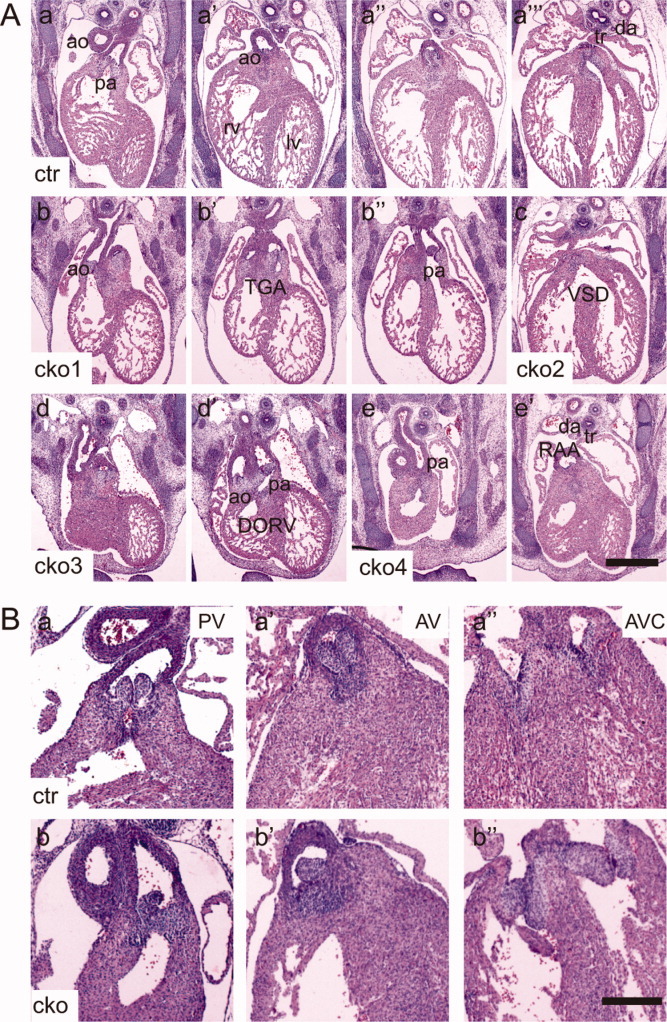
Heart defects in *Sur-8*^Δ/flox^;Tie2-Cre embryos. **A:** Microscopic images showing representative transverse sections from the control (a–a″′) and four *Sur-8*^Δ/flox^;Tie2-Cre (b–e′) E13.5 embryos. The images of the sections were arranged from anterior to posterior for each embryo. Note the following heart defects: transposition of the great arteries (TGA, b–b″); ventricular septal defect (VSD, c); double outlets of the right ventricle (DORV, d and d′) and right aortic arch (RAA, e and e′). ao, aorta; pa, pulmonary artery; rv, right ventricle; lv, left ventricle; tr, trachea; da, dorsal aorta. Scale bar = 0.5 mm. **B:** Images of representative transverse sections from the control and mutant E14.5 embryos. a–a″: Symmetrical outflow tract valves (pulmonary and aortic valves) and mature thinly tapered atrioventricular valves (tricuspid and mitral valves) in the control embryos. b–b″: Outflow tract valve with asymmetrical structure and the underdeveloped atrioventricular valve leaflets in *Sur-8*^Δ/flox^;Tie2-Cre hearts. PV, pulmonary valve; AV, aortic valve; AVC, atrioventricular canal. cko, *Sur-8*^Δ/flox^;Tie2-Cre; ctr, *Sur-8*^Δ/flox^. Scale bar = 0.8 mm.

To examine the possible cardiac defects in the mutant embryos, embryonic hearts were serially sectioned for histological analysis. Compared to the control embryos, the *Sur-8* CKO embryos showed a range of cardiac defects at E13.5 and E14.5, including ventricular septal defects (VSD), double-outlet right ventricle (DORV), transposition of the great arteries (TGA) and right aortic arch (RAA) (Fig. 2A, Table 2).

**TABLE 2:** Cardiac Phenotypes in *Sur-8*^Δ/*flox*^; *Tie2-Cre* Embryos

Embryonic stage	#embryos	VSD	TGA	DORV	RAA
E13.5	12	12	1	3	4
E14.5	13	12	0	3	2

Abbreviations: VSD, ventricular septal defect; RAA, right aortic arch; DORV, double-outlet right ventricle; TGA, transposition of the great arteries.

Furthermore, the defects of heart valve morphogenesis were found in *Sur-8* CKO embryos. For example, the outflow tract valves in all the *Sur-8* CKO embryos examined were asymmetrically aligned, differing from that of the controls (Fig. 2B); the atrioventricular (AV) valves had mature thinly tapered leaflets in the normal hearts, whereas 7 out of 13 *Sur-8* CKO hearts were underdeveloped with enlarged bulbous primordial leaflets (Fig. 2B). These data indicate that valvular development was impaired in *Sur-8* CKO embryos.

### SUR-8 Is Required for Normal Cellularization of AV Cushions

**Fig. 3:**
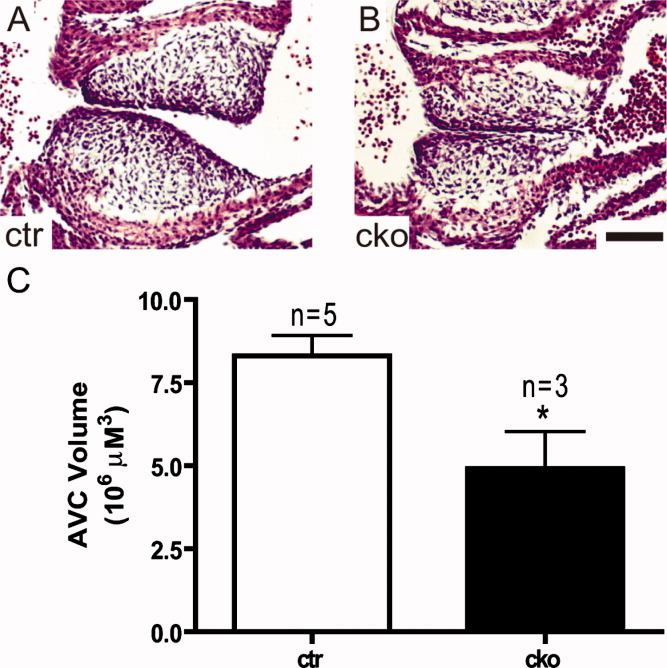
Hypoplastic AV cushions in *Sur-8*^Δ/flox^;Tie2-Cre embryonic heart. H&E-stained histological sections of the middle portion of the atrioventricular canal showing the underdeveloped cushions in E11.5 *Sur-8*^Δ/flox^;Tie2-Cre embryos (**B**) compared to that in controls (**A**). Scale bar = 100 μm. **C:** Stereologic analysis showing smaller cushion volume of *Sur-8*^Δ/flox^;Tie2-Cre embryos compared with that of the controls. Data shown as mean ± SEM. *P < 0.05, by 2-tailed Student's *t*-test. cko, *Sur-8*^Δ/flox^;Tie2-Cre; ctr, *Sur-8*^Δ/flox^.

Vertebrate valve formation is characterized by the initial formation of endocardial cushions in the atrioventricular canal (AVC) and outflow tract, followed by the proliferation of heart valve progenitor cells and the remodeling of primodial valves (Schroeder et al.,^2003^; Armstrong and Bischoff,^2004^; Person et al.,^2005^; Yutzey et al.,^2005^). To evaluate the development of endocardial cushions, we measured cushion volumes at E11.5, just as cushions complete endothelial-mesenchymal transformation (EMT) and undergo the proliferation phase. The result of this analysis indicated that the *Sur-8* CKO atrioventricular cushions were 40.8% smaller (*P* < 0.05) than that of the controls (Fig. 3). The average cell density of AV cushions at E11.5 was comparable between the *Sur-8* CKO AVCs and controls (data not shown). In contrast, the outflow tract cushions did not show a significant difference between the *Sur-8* CKO hearts and that of the controls (Fig. S2).

### SUR-8 Is Necessary for AVC Endothelial-Mesenchymal Transformation

**Fig. 4:**
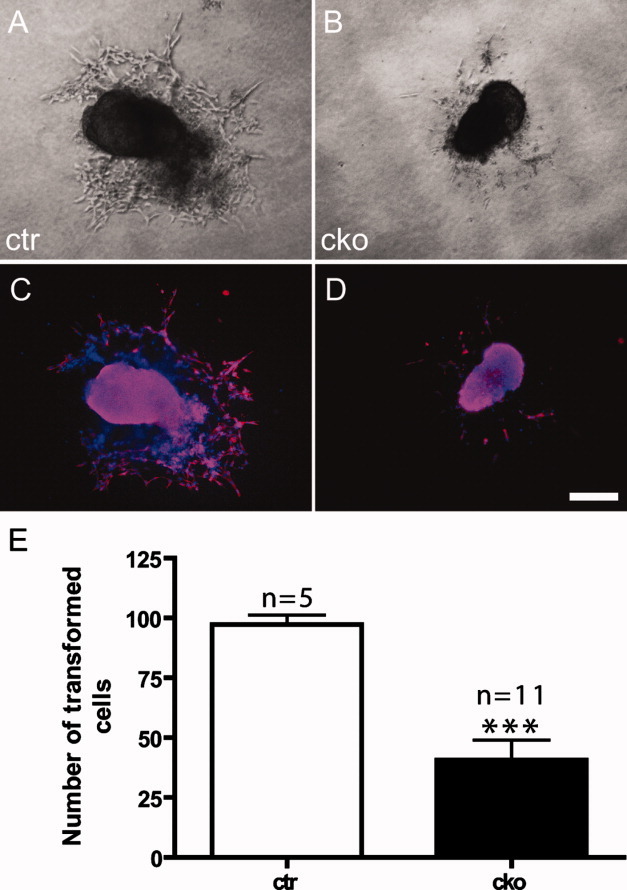
SUR-8 is required for proper AV cushion EMT in vitro. **A, B:** Images showing AVC explants from control (ctr) and *Sur-8*^Δ/flox^;Tie2-Cre (cko) embryos at E9.5 seeded onto type I collagen gels in an in vitro collagen gel analysis. The endocardium of *Sur-8*^Δ/flox^; Tie2-Cre explants did not show the expansion of transformed mesenchymal cells to a similar extent as did the control explants. **C, D:** Immunofluorescence images of the same explants in A and B, stained using an antibody against α-smooth muscle actin (α-SMA, red). Total nuclei were visualized with DAPI staining (blue). Fewer α-SMA-positive cells were observed in mutant explants (D) than in controls (C), confirming the reduction of transformed cells in *Sur-8*^Δ/flox^;Tie2-Cre explants. Scale bar = 20 μm. **E:** Diagrams showing quantitative data of the number of transformed cells in both control and *Sur-8*^Δ/flox^; Tie2-Cre explants counted under a light microscope. Data shown as mean ± SEM. ***P < 0.0001, by 2-tailed Student's *t*-test.

In the beginning of endocardial cushion formation, a subpopulation of endothelial cells within the atrioventricular canal undergoes an important process called endothelial-mesenchymal transformation (EMT), featuring obvious morphological changes, such as loss of cell-cell adhesions, and extension of filopodia into the extracellular matrix to separate the endocardium from the myocardium (Markwald et al.,^1979^). In vitro collagen gel analysis was performed on the dissected cushion explants to test whether SUR-8 is required for EMT. This assay was first established to study EMT in the AVC region of chicken embryonic hearts (Bernanke and Markwald,^1982^), and has recently been applied to study mouse EMT (Camenisch et al.,^2002^). The AVC endothelial cells of *Sur-8* CKO explants underwent a smaller extent of expansion compared with well-formed mesenchymal cells in controls (Fig. 4A, B). Immnofluorescent staining using an antibody against α-smooth muscle actin, widely used as the cushion mesenchymal marker (Nakajima et al.,^1997^; Sugi et al.,^2004^), confirmed that less mesenchymal cells were detected in the *Sur-8* CKO explants compared to that of controls (Fig. 4C, D). Quantitative analysis showed that the number of transformed mesenchymal cells in *Sur-8* CKO explants was reduced to ∼50% of controls (Fig. 4E), indicating that SUR-8 plays a critical role in EMT.

### SUR-8 Is Required for Normal Proliferation of Mesenchymal Cells

**Fig. 5:**
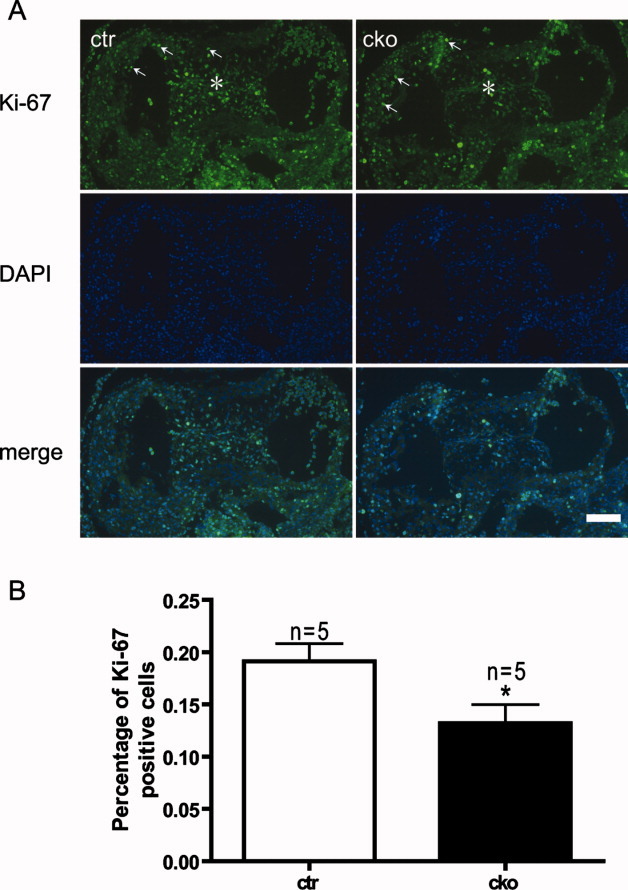
Reduction of cell proliferation in AV cushion of *Sur-8*^Δ/flox^;Tie2-Cre embryonic hearts. **A:** Fluorescence images showing proliferating cells identified by Ki-67 immunofluorescence staining in sections of control (ctr) and *Sur-8*^Δ/flox^;Tie2-Cre (cko) atrioventricular endocardial cushions. Total nuclei were stained with DAPI. *Indicates the stained AV cushions, and Ki-67-positive nuclei were also observed in myocardium (white arrows). **B:** Diagram showing the average percentage of proliferating cells within the cushions. Data were averaged from five embryos for each genotype, and at least 400 nuclei were counted for each embryo. Data shown as mean ± SEM. *P < 0.05, by 2-tailed Student's *t*-test. Scale bar = 100 μm.

After EMT, transformed mesenchymal cells undergo several rounds of cell division to expand the endocardial cushion. To evaluate the proliferative status of the mesenchymal cells, staining with the Ki-67 antibody, which labels proliferating cells, was performed on the sections of embryonic hearts (Fig. 5A). The average ratio of Ki-67-positive cells was significantly reduced in *Sur-8* CKO AVCs at E11.5 compared to that of controls (19.1±1.7 versus 13.2±1.8%, *P* < 0.05, Fig. 5B), indicating that SUR-8 is required for the proliferation of transformed mesenchymal cells. Apoptotic cell death occurred neither in *Sur-8* CKO at E11.5 nor in controls (data not shown).

### SUR-8 Deficiency in Endothelial Cells Does Not Alter Activation of ERKs

**Fig. 6:**
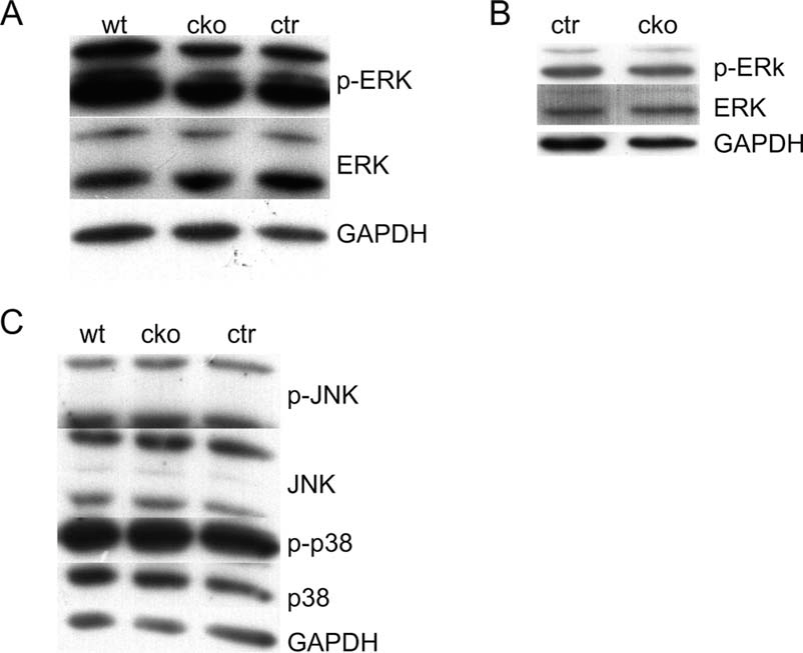
ERKs activation is not affected in either *Sur-8*^Δ/flox^;Tie2-Cre endothelial cells or AV cushions. **A, B:** Western blot analysis of activation of ERK in endothelial cells isolated from E11.5 embryos (A) and whole AV cushion lysates (B). **C:** Western blot analysis of activation of JNK and p38 kinases in isolated endothelial cells. No significant alterations were detected in A–C. GAPDH was used as an internal control. cko, *Sur-8*^Δ/flox^;Tie2-Cre; ctr, *Sur-8*^Δ/flox^; wt, *Sur-8*^flox/+^.

SUR-8 has been proposed to function as a scaffold-like protein that facilitates an interaction between Ras and Raf, and previous genetic data support its role as a positive regulator of the Ras signaling pathway (Sieburth et al.,^1998^; Li et al.,^2000^). Mutating *Sur-8* in endothelial cells might compromise the Ras-Raf-ERK signaling pathway, and lead to the defects described above. To examine the effect of SUR-8 inactivation on the Ras-Raf-ERK pathway, activated ERK levels (cellular readout for Ras-Raf signaling pathway) in isolated endothelial cells from E11.5 embryos was measured by Western blotting using an antibody specific for phosphorylated ERK. Surprisingly, there was no obvious change in the level of activated ERK in *Sur-8* CKO endothelial cells compared to that of controls (Fig. 6A). Samples derived from E11.5 AVC cushion tissue also failed to show an obvious change in ERK activation (Fig. 6B). The activation level of two other MAP kinases, JNK and p38, did not show any difference between the endothelial cells derived from E11.5 CKO mutant embryos and the controls (Fig. 6C). These results indicate that SUR-8 deletion does not alter ERK activation in the developing AVC cushions.

## DISCUSSION

In this study, we characterized the developmental defects in the *Sur-8*^Δ/flox^;Tie2-Cre mouse embryo, and found that SUR-8 deficiency in the vascular system led to multiple cardiac defects and late embryonic lethality. Moreover, we showed that the formation of endocardial cushions was impaired in the atrioventricular canal of the *Sur-8* CKO hearts, mainly due to reduced EMT. These data present the first analysis of physiological function of SUR-8 using mammalian genetics and provide valuable insights about its essential role in valvulogenesis. More significantly, our results implicate a novel function for SUR-8 that is independent of the activation of MAP kinases, which deviates from the known function of SUR-8 in promoting Ras-Raf-ERK signaling supported by previous studies in *C. elegans* and mammalian tissue culture cells.

The gross network of the peripheral vessels in *Sur-8*^Δ/flox^;Tie2-Cre embryos appeared to develop normally. However, the mutant embryos died at or after E13.5, and 50% of them showed dorsal edema, indicating that the *Sur-8* CKO embryos may suffer from heart failure. We confirmed this prediction by detailed histological examination. Multiple cardiac defects including ventricular septal defect (VSD), doubling of the right ventricles (DORV), transposition of the great arteries (TGA) and the right aortic arch (RAA) were observed in the mutant hearts. Among these defects, we noticed that VSD occurred in almost every mutant embryo examined, whereas the other three malformations were sporadic events. VSD may be caused by defects in the cardiac cushions and valves, which are responsible for heart septation. Abnormal morphogenesis in cardiac cushion and valvular defects were indeed observed in the CKO embryos. Intriguingly, about half of the CKO hearts displayed underdeveloped AV valves with enlarged leaflets at E14.5. Given that the AV cushions need to undergo the final remodeling process to form mature thin tapered leaflets, it is likely that SUR-8 may also be required for the successful remodeling of heart valves. Due to the fact that the etiology of cardiovascular malformations often involves both genetic causes and hemodynamic effects (Yashiro et al.,^2007^), the exact causes of these structural defects remain to be investigated.

EMT is a critical step during endocardial cushion formation. Reduced EMT contributed to the hypoplastic atrioventricular cushions in *Sur-8* CKO hearts. However, neither outflow tract volume nor EMT was affected in the mutant hearts (Supp. Figs. S2 and S3). The different effects observed between the outflow tract and AV cushions may reflect the inherent and developmental differences between the two sets of cushions. In addition to their different positions within the developing heart, the outflow tract and AV cushions also differ with respect to their cellular composition. For AVC, the final valves are largely derived from endocardial cushions (Wessels et al.,^1996^). However, the development of the outflow tract valves is more complex. In addition to the formation of outflow tract proximal cushions from the outflow tract endothelium, a population of neural crest cells derived from branchial arches migrate to the mid and distal outflow tract to form intact outflow tract cushions (Stoller and Epstein,^2005^). Furthermore, the timing of EMT initiation is different between AV and outflow tract cushions. AV cushion EMT initiates at about E9.5, while outflow tract EMT appears to be delayed by approximately one day in mouse (Camenisch et al.,^2002^). Thus, it is possible that by E10.5, other redundant signaling factors may compensate for the loss of SUR-8 to ensure normal outflow tract EMT in CKO mutants.

SUR-8 had been demonstrated to modulate ERK activation by regulating the interaction between Ras and Raf (Selfors et al.,^1998^; Sieburth et al.,^1998^; Li et al.,^2000^). To our surprise, no differences in ERK phosphorylation status were detected in either the *Sur-8* CKO endothelial cells or the atrioventricular cushion tissues by Western blotting (Fig. 6A, B). We also failed to detect activated ERK in endothelial cells of endocardial cushion sections at E9.5 and E10.5 from wild-type mice by immunostaining (data not show), which is consistent with the published observation that activated ERK is maintained at a very low level in the normal physiological context (Gitler et al.,^2003^). This low level would thus prevent us from further comparing the activated ERK levels between the sections derived from the CKO and control cushions. The results obtained from this study suggest that SUR-8 may act in an ERK-independent regulatory pathway for its essential function in valvulogenesis. In supporting this possibility, we also observe no significant change in the expression level of the transcription factor snail1 (Supp. Fig. S4), which has been shown to be induced by the ERK pathway and plays a fundamental role in the repression of E-cadherin during EMT (Delfini et al.,^2009^; Thiery et al.,^2009^). However, we could not exclude the possibility that SUR-8 still plays a role in ERK activation and the activity of ERK in CKO mutants is altered in a subtle manner or in a narrow window of time.

Multiple signaling pathways and transcription factors integrate to coordinately regulate EMT and cushion formation (Armstrong and Bischoff,^2004^). Functional analysis of EGF-family ligands and receptors through targeted mutations in mice provided much evidence about the contributions of altered EGF signaling to defective valvulogenesis (Schroeder et al.,^2003^). Unlike hyperplastic cushions observed in heparin-binding (HB)-EGF or EGFR mutant hearts (Iwamoto et al.,^2003^; Jackson et al.,^2003^), mice lacking ErbB3 died at midgestation due to the hypoplastic AV cushion defect (Erickson et al.,^1997^), implying that SUR-8 might act downstream of ErbB3 signaling. We also examined the expression level of some well-known transcription factors involved in EMT and atrioventricular (AV) cushion formation. Snail1 is a transcription factor downstream of the Notch signaling pathway and functions as a repressor of E-cadherin to modulate EMT of endothelial cells (Timmerman et al.,^2004^). Smad6 is a repressor of BMP signaling and reduces EMT (Galvin et al.,^2000^; Desgrosellier et al.,^2005^). hHex is a homeobox transcription factor, regulating EMT by repressing Vegfa level (Hallaq et al.,^2004^). Among the GATA family of the zinc finger transcription factors, GATA4, 5, and 6 are expressed in the developing heart and are required for heart formation (Heikinheimo et al.,^1994^; Charron and Nemer,^1999^). Only GATA4 expression was shown to be significantly reduced (Fig. S4), indicating a specific regulatory role of SUR-8 on GATA4 expression. In humans, GATA4 haploinsufficiency causes severe defects in valve development (Garg et al.,^2003^; Okubo et al.,^2004^). Although GATA4 deficiency in mice would lead to hypocellular AV cushions and embryonic death, the heterozygous GATA4 mutant mice developed normally (Rivera-Feliciano et al.,^2006^). In addition to being regulated at the transcription level, the transcription factors involved in EMT could also be modified at posttranslational levels (Thiery et al.,^2009^). Thus, we hypothesize that in the SUR-8 CKO mice, the impairment of AVC EMT may be caused by the combination of alterations in the expression or activities in GATA4 and other factors. Analysis of genome-wide gene expression or protein modification in CKO mutants may help to elucidate how SUR-8 regulates EMT and AV cushion formation during embryonic heart development.

## EXPERIMENTAL PROCEDURES

### Targeting Vector Construction and Animals

The targeting vector for the *Sur-8* gene was constructed from a 129 genomic DNA fragment screened out of a BAC library (Invitrogen, Carlsbad, CA). The XbaI-XbaI fragment within intron 2, the XbaI-BglII fragment containing exon 3, and the BglII-SpeI fragment within intron 3 from the *Sur-8* gene were used as the long arm, center fragment, and short arm in the targeting vector, respectively. These three fragments were inserted into the relative positions of plasmids containing *loxP* sites and a neomycin-resistant cassette flanked with *frt* sites to construct the targeting vector (Fig. 1A). The targeting construct was introduced into W4/129S6 embryonic stem cells (Taconic) by electroporation. After double selections, positive clones were screened by PCR and further verified by Southern blot. Chimeric mice were obtained from two independent positive ES clones. Genomic DNA was extracted and genotyped using a three-primer PCR analysis. Primer sequences for *Sur-8* CKO genotyping were available upon request.

Animal-related procedures were reviewed and approved by the Animal Care and Use Committee of the Institute of Developmental Biology and Molecular Medicine at Fudan University, Shanghai, People's Republic of China.

### Isolation of Endothelial Cells

Embryos from E11.5 were treated in HBSS (Gibco, Gaithersburg, MD; Ca^++^/Mg^++^) with 200 U/ml collagenase IV (Gibco) and 10 μg/ml DNase I for 45 min at 37°C. To purify endothelial cells, cells pellets were resuspended in washing buffer (2 mM EDTA in PBS, Ca^++^/Mg^++^ free, pH 7.4) with 2 μg/ml PECAM-1 antibody (PharMingen, San Jose, CA; 558736) and incubated for 30 min at 4°C. They were then incubated with Dynabeads (Dynal, Carlsbad, CA; sheep anti-rat IgG dynabeads) with rocking for an additional 30 min at 4°C. Bead-bound endothelial cells were separated by a magnet (Dynal MPC-L) in washing buffer for a further application.

### RT-PCR Analysis

Total RNA from endothelial cells was extracted with RNeasy Mini Kit (Qiagen, Chatsworth, CA) and treated with RNase-free deoxyribonuclease I (Takara Bio, Shiga, Japan) to eliminate genomic DNA contamination. Reverse transcription PCR was carried out with AWV RNA PCR Kit (Takara) according to the manufacturer's instructions. Real-time PCR was performed with 2× HotSybr PCR Reaction Mix (NuStar Laboratory) on an Mx3000P Quantitative PCR System (Stratagene, La Jolla, CA) following the manufacturer's instructions. Expression of GAPDH was used as the baseline standard for real-time PCR.

### Western Blotting

Total protein extracted from isolated endothelial cells and AV cushion tissues were analyzed by Western blotting following protocols described previously (Tian et al.,^2008^). We used the following primary antibodies: anti-SUR-8/SHOC2 (HPA009164, Sigma, St. Louis, MO; 1:500), anti-phospho-Erk1/2 (9101, Cell Signaling Technology, Danvers, MA; 1:1,000), anti-Erk1/2 (9107; 1:2,000), anti-phospho-JNK (9251; 1:1,000), anti-JNK (9252; 1:1,000), anti-phospho-p38 (9215; 1:1,000), anti-p38 (9212; 1:1,000), and anti-GAPDH (KC-5G4, KangCheng Biotech, Shanghai, PR China; 1:10,000). HRP-conjugated goat anti-mouse (1:4,000), or goat anti-rabbit (1:4,000) secondary antibodies (Santa Cruz Biotechnology, Santa Cruz, CA) were used to visualize the specific bands.

### Histological Analysis and Cushion Volume Measurements

Paraffin sectioning and frozen sectioning were performed as described previously (Zhang et al.,^2007^). Paraffin sections were stained with hematoxylin and eosin (H&E). Frozen sections were applied in antibody staining assay. Immunofluorescence was performed as described previously (Song et al.,^2007^). Ki-67 (NCL-Ki67p, Novocastra, Bannockburn, IL; 1:1,000) was used as a cell proliferation marker and goat anti-rabbit IgG-FITC (Sigma) was used as the secondary antibody. Total nuclei were visualized with DPAI staining. Photographs were taken using a Leica DMRXA2 system equipped with A and GFP filters and a Leica DFC300 CCD camera. The cushion volume measurement was analyzed as described (Stephen et al.,^2007^).

### Collagen Gel Assay for Cushion Mesenchyme Formation

Endocardial explants were cultured as previously described (Camenisch et al.,^2002^). Migrating mesenchymal cells were identified by α-SMA antibody (Sigma, A5228) staining, which was performed as described (Hallaq et al.,^2004^). Nuclei were stained with DAPI. Images were acquired by a Leica DMIRE2 system equipped with A and GFP filters and recorded by a Leica DC350F CCD camera. Endocardial cell transformation was assessed by counting the number of cells that invaded the collagen matrix as well as the number of migrating cells on the collagen gel surface. The data were analyzed using Student's *t*-test (significance was set at *P* < 0.05).

## Supplementary material

Supporting Figure S1: Vascular integrity in Sur-8Δ/flox;Tie2-Cre embryos. A: E14.5 mutant (cko) embryos showing severe dorsal oedema (arrowhead) compared to control embryo. B: H&E-stained histological sections showing congestion in the lobes of fetal lungs in cko embryos. All mutant embryos examined (n=13) show this defect at E14.5. C: Whole-mount PECAM-1 staining showing similar peripheral vascular system between cko and control embryos at E10.5. cko, Sur-8Δ/flox;Tie2-Cre; ctr, Sur-8Δ/flox. Scale bar = 1 mm for A and C, 0.3 mm for B.

Supporting Figure S2: The outflow tract cushion volume in Sur-8Δ/flox;Tie2-Cre is not significantly different from that in the control hearts. A, B: H&E-stained histological sections through the middle part of outflow tract cushions of E11.5 controls and mutant (cko) hearts. C: The average volume of outflow tract endocardial cushions in the mutants is slightly lower but still comparable to that in the controls. cko, Sur-8Δ/flox;Tie2-Cre; ctr, Sur-8Δ/flox. Scale bar = 0.1 mm.

Supporting Figure S3: The outflow tract cushion EMT in Sur-8Δ/flox;Tie2-Cre mice is similar to that in the controls. A, B: Microscopic images showing the results of collagen gel assay performed with outflow tract explants of mice at E10.5. Similar EMT patterns were observed between the control and the mutant (cko) explants after 48 hr culturing. C, D: Fluorescence images showing the migrating mesenchymal cells stained by antibody against α-smooth muscle actin (red). cko, Sur-8Δ/flox;Tie2-Cre; ctr, Sur-8Δ/flox. Scale bar = 20 μm.
