# Supplementary figures and images for: Endothelial SUR-8 Acts in an ERK-Independent Pathway During Atrioventricular Cushion Development
Source: Dev Dyn. 2010 Jun 14;239(7):2005–13. doi: 10.1002/dvdy.22343 (PMC3138404; doi:10.1002/dvdy.22343)

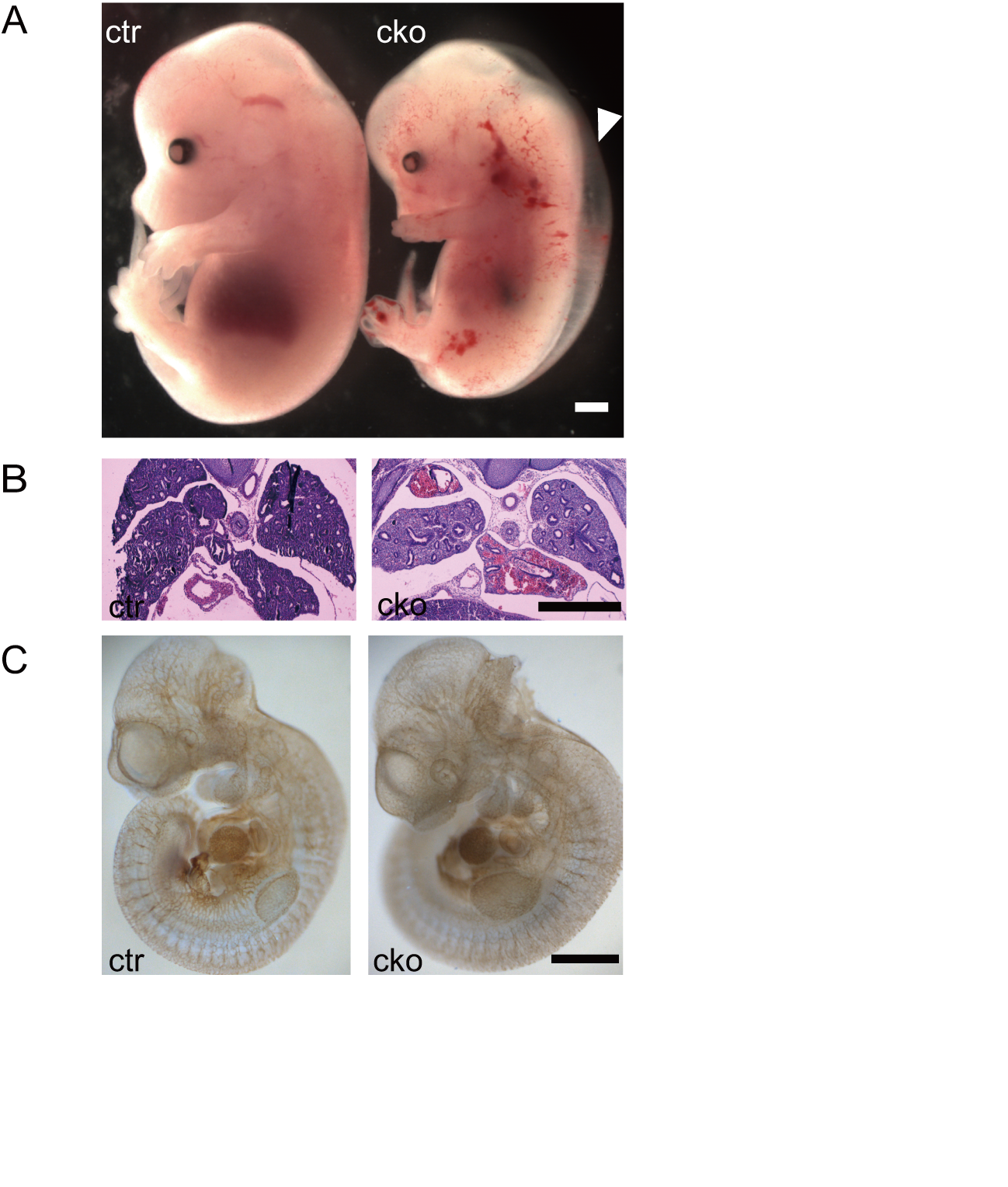

Supplement: Supplementary file 1 [file dvdy0239-2005-SD1.tif]

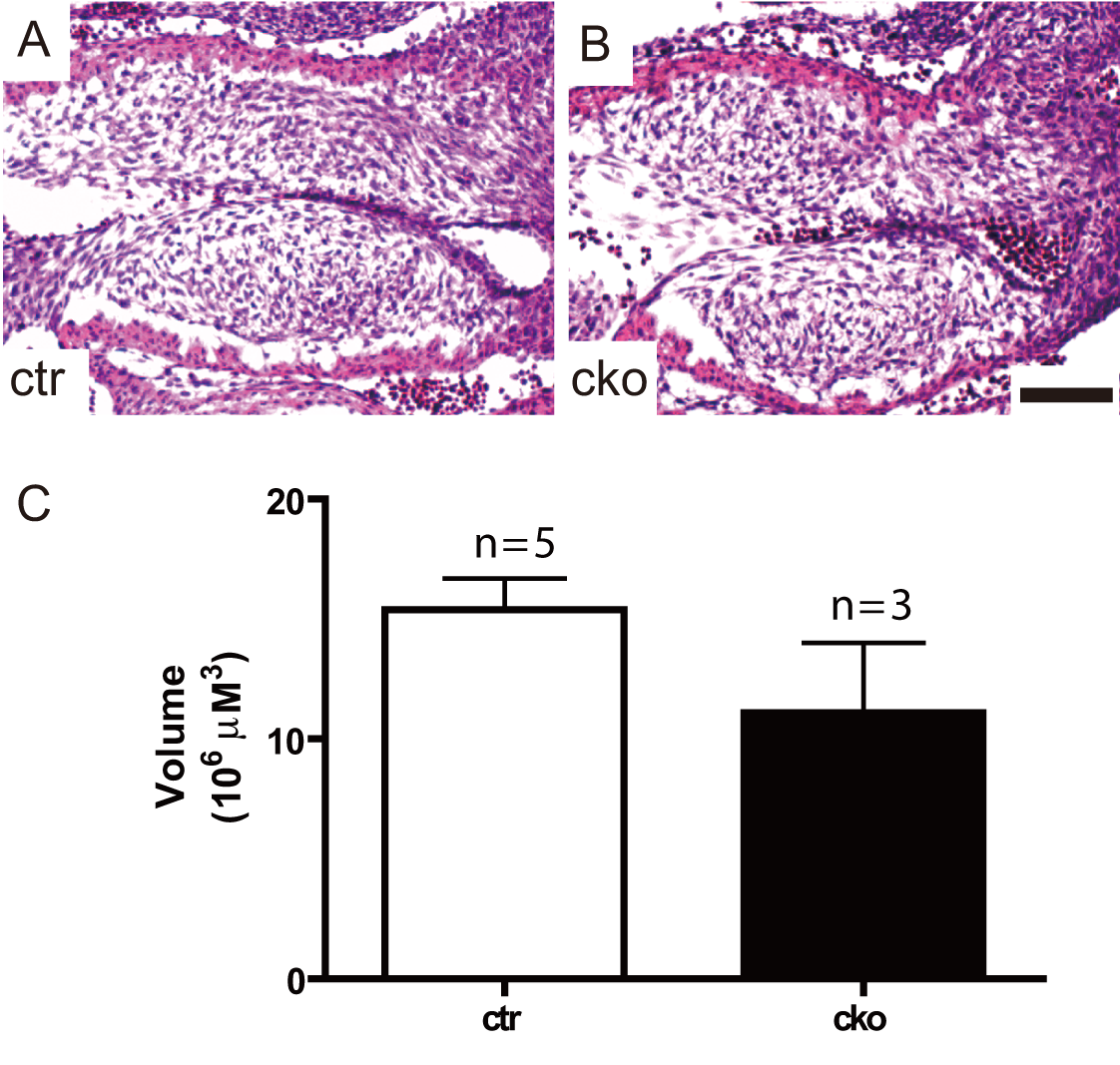

Supplement: Supplementary file 2 [file dvdy0239-2005-SD2.tif]

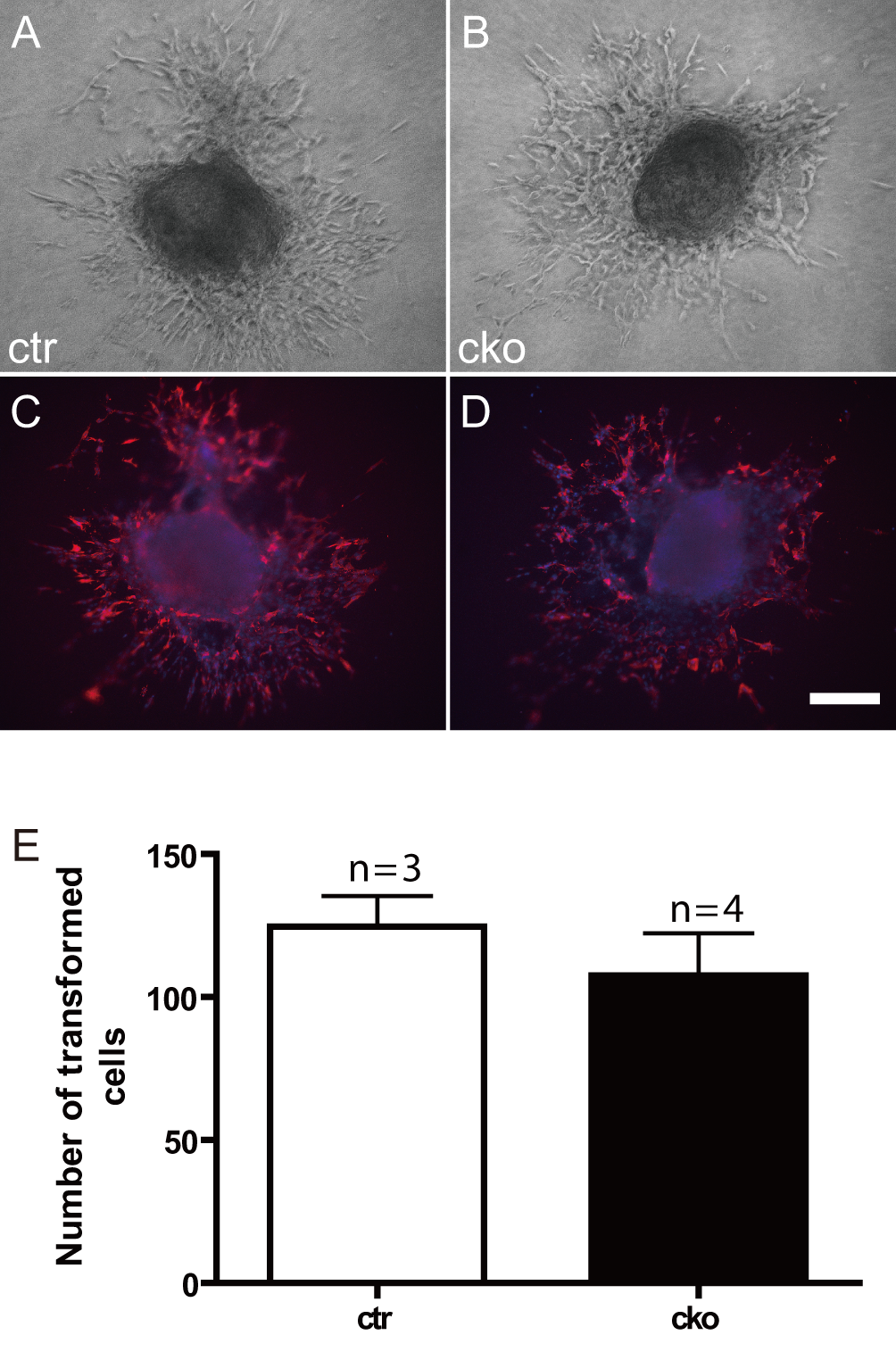

Supplement: Supplementary file 3 [file dvdy0239-2005-SD3.tif]

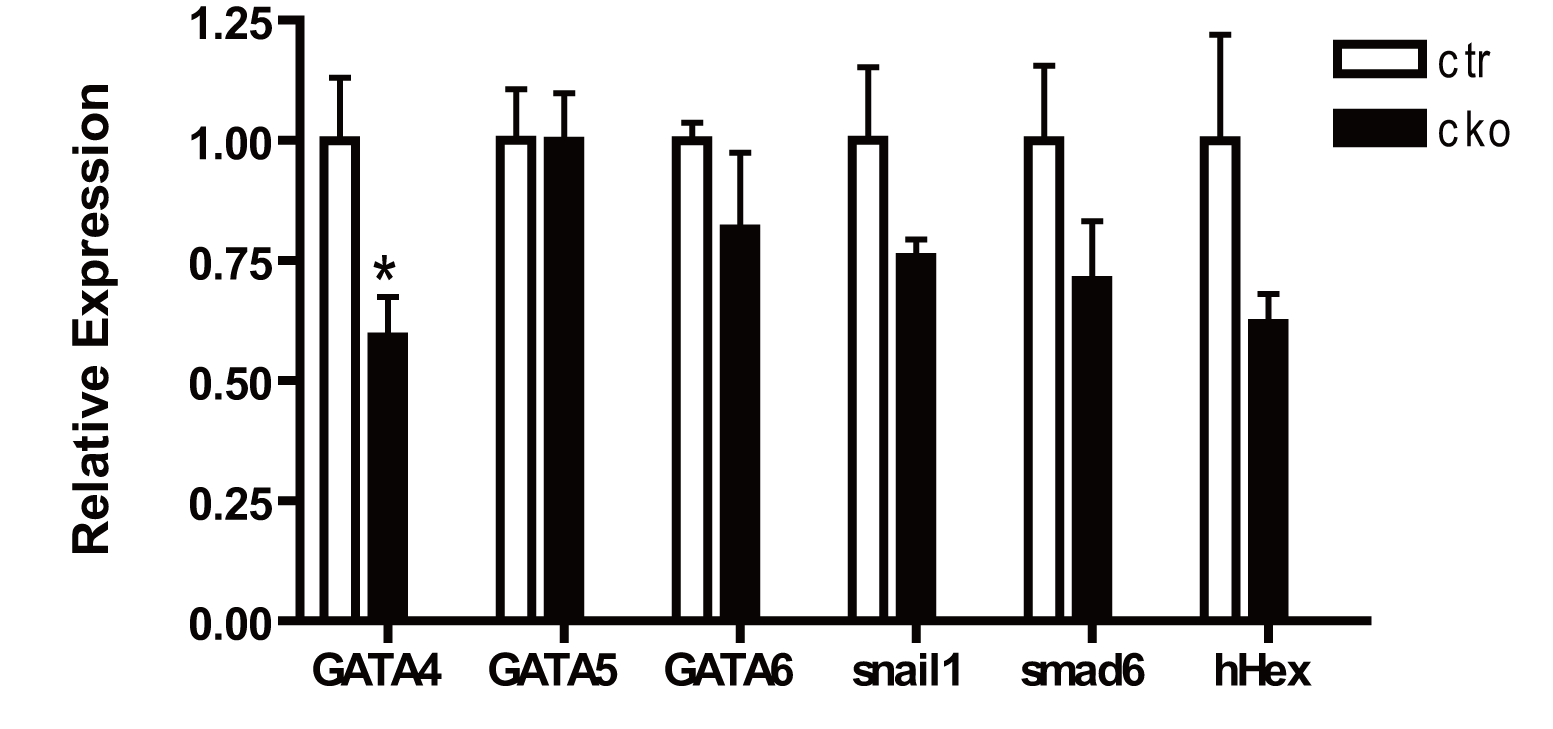

Supplement: Supplementary file 4 [file dvdy0239-2005-SD4.tif]
